# Mesoscopic packing of disk-like building blocks in calcium silicate hydrate

**DOI:** 10.1038/srep36967

**Published:** 2016-11-15

**Authors:** Zechuan Yu, Ao Zhou, Denvid Lau

**Affiliations:** 1 Department of Architecture and Civil Engineering, City University of Hong Kong, Hong Kong, China; 2 Department of Civil and Environmental Engineering, Massachusetts Institute of Technology, Cambridge, MA 02139, USA

## Abstract

At 100-nanometer length scale, the mesoscopic structure of calcium silicate hydrate (C-S-H) plays a critical role in determining the macroscopic material properties, such as porosity. In order to explore the mesoscopic structure of C-S-H, we employ two effective techniques, nanoindentation test and molecular dynamics simulation. Grid nanoindentation tests find different porosity of C-S-H in cement paste specimens prepared at varied water-to-cement (w/c) ratios. The w/c-ratio-induced porosity difference can be ascribed to the aspect ratio (diameter-to-thickness ratio) of disk-like C-S-H building blocks. The molecular dynamics simulation, with a mesoscopic C-S-H model, reveals 3 typical packing patterns and relates the packing density to the aspect ratio. Illustrated with disk-like C-S-H building blocks, this study provides a description of C-S-H structures in complement to spherical-particle C-S-H models at the sub-micron scale.

Calcium silicate hydrate (C-S-H) gel is a major hydration product in cement. In decades of studies, a lot of information about the microstructure of C-S-H has been collected and translated into theoretical descriptions and numerical models. Nowadays, the challenge is to link the C-S-H structure at the microscopic scale to the material properties at the macroscopic scale^1^,^2^. Reviewing the literature, the material properties and the microstructure of C-S-H can be elaborated in a multi-scale manner^3^. At the atomistic level (*i.e.* 0.1 to 10 nanometers), crystals such as tobermorite, jennite and recently clinotobermorite are used as the starting point for constructing atomistic C-S-H models^4^,^5^,^6^. At this length scale, C-S-H nanoparticle is described as a layered structure consisting of one mineral-rich layer and one intra-particle water layer alternatively^7^,^8^. Several C-S-H layers can form a branch-like nanostructure^9^, connected with each other via electrostatic and van der Waals forces. By performing molecular dynamics simulation and further numerical analysis, one can measure material properties of C-S-H nanocrystals and adhesion strength between C-S-H layers^10^,^11^,^12^. The internal structural water is found to be a scaffolding component that enhances the mechanical properties of C-S-H^13^. The Young’s modulus of C-S-H nanoparticles is around 70 GPa^14^, higher than that of conventional concrete. This information implies that cement-based materials are of great potential and could be further upgraded by fine-tuning the nano- and microstructure, such as by implanting cementitious nanotubes^15^,^16^. Up to the sub-micron level (*i.e.* 10 to 1000 nanometers), it is generally accepted that C-S-H is made up of small building blocks with a characteristic length of around 4–5 nm^17^,^18^. These basic units can flocculate to form high-density (HD) or low-density (LD) C-S-H depending on packing density^19^,^20^,^21^. At this length scale, C-S-H can be described as a system of cohesive rigid objects. The modulus of HD C-S-H is around 30 GPa, not as good as individual C-S-H nanoparticles. The degraded mechanical performance could be ascribed to sub-micron pores and cavities. Essential structural characteristics, including specific surface area, pore size and volume fraction, can be measured by experimental techniques such as small angle neutron scattering (SANS) and small angle X-ray scattering (SAXS)^7^,^22^. Recent experiments have observed the existence of disk-like C-S-H building blocks^23^. At the macroscopic scale (*i.e.* over 1 micrometer), nanoindentation tests can measure the hardness and modulus of C-S-H in cement pastes. Statistical analysis on the nanoindentation measurements further provides information about packing density and phase composition of constitutes in the indented material^24^. Assuming that C-S-H is composed of dense mineral core and gel pores, it has been found that all C-S-H phases contain a mineral core with the same mechanical properties^25^. The mineral phase is similar to the previously mentioned C-S-H nanoparticles in mechanical properties, showing the consistency between nanoindentation technique and atomistic modeling works. On the basis of the concept of building blocks or nanoparticles, mesoscopic models of the C-S-H aggregation can be proposed. A discrete element method has been employed in modeling nanoindentaion tests on C-S-H specimens with varied packing factors^26^. The simulated nanoindentation tests have shown a good agreement with experiments. The sub-micron structure of C-S-H has been described by a colloidal model^27^, which does not capture the lamellar nature of silica skeletons in C-S-H. As an extension, modeling of disk-like objects can reflect the morphology of the solid silica layers. The concept of C-S-H disk has been employed in the framework of continuum micromechanics to demonstrate the early strength development of C-S-H^28^. Column-like and porous polycrystalline structures have been built and investigated with the continuum models^28^,^29^. Here, a mesoscopic molecular dynamics model is developed using the concept of disk-like objects^11^,^30^,^31^. Simulations using this model could embody the lamellar nature of C-S-H gel at the scale of around 100 nanometers.

This study aims to link the structure of C-S-H at the sub-micron scale to the material properties at the macroscopic scale. Grid nanoindentation tests are employed to measure the material properties and molecular dynamics simulations are performed to reproduce the structures of C-S-H. The nanoindentation tests, with statistical analysis, find that C-S-H in cement pastes prepared at different w/c ratios show different phase compositions and porosities. Considering that the porosity could be related to the structure of C-S-H, we develop a mesoscopic model and perform molecular dynamics simulations to investigate how the w/c ratio induces changes to the C-S-H structure and affects the gel porosity. This study provides illustrations of the mesoscopic structure of C-S-H, links the C-S-H structure to the porosity and could enrich our understandings of cement-based materials at small scales.

## Results

### Deconvolution of nanoindentation data

**Figure 1:**
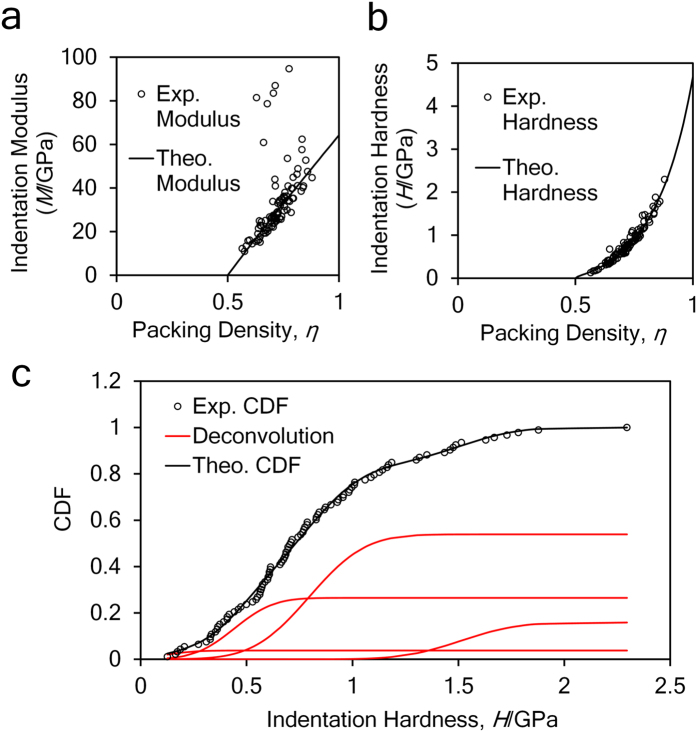
(**a,b**) Calculation of packing density on the basis of indentation modulus and hardness information. (**c**) The deconvolution of hardness results. This is a sample deconvolution result obtained from 93 nanoindentation tests on cement paste with 0.3 w/c ratio.

**Figure 2:**
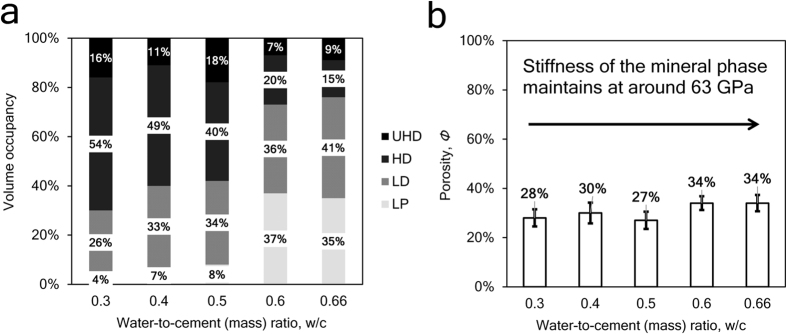
(**a)** Volume occupancy of C-S-H phases in cement samples. (**b**) The porosity of cement samples with varied water-to-cement ratios.

The plots of indentation modulus, indentation hardness and the packing density are shown in Fig. 1a and b. In Fig. 1a, some data points lie away from the theoretical modulus line, which is a commonly observable phenomenon and it implies that the empirical formulas violate for some indentation tests^24^,^25^. The relative errors of reconstructed indentation hardness and indentation modulus are around −5%, the standard deviations are around 20%, within the acceptable range^25^. A sample deconvolution result obtained from 93 tests on the cement paste with 0.3 w/c ratio is shown in Fig. 1c. The deconvolution error is in the order of 10^−4^, within an acceptable range. Deconvolution results of volume fractions of 4 characteristic C-S-H phases and indentation properties are listed in Table 1. The indentation moduli of the four characteristic phases (LP, LD, HD and UHD C-S-H) are around 10 GPa, 20 GPa 30 GPa and 50 GPa respectively, comparable to existing studies^25^. The cement sample with w/c = 0.3 contains 4% LP phase, 26% LD phase, 54% HD phase and 16% UHD phase, in good agreement with previous grid nanoindentation results^24^. As the w/c ratio increases, more low-density phases (LP + LD) are found and the porosity of the entire C-S-H composite increases, as plotted in Fig. 2. With the increase of w/c ratio, the increase of porosity is a common phenomenon observed from experiments^20^,^32^,^33^ and simulations^34^. The stiffness of the mineral phase ranges from 62 GPa to 64 GPa in all the samples, comparable to previous studies^25^. The similar mineral properties in turn indicate that the grid nanoindentation results are performed on a group of materials with similar mechanical behaviors.

### Change of porosity predicted by mesoscopic models with varied building block sizes

**Figure 3:**
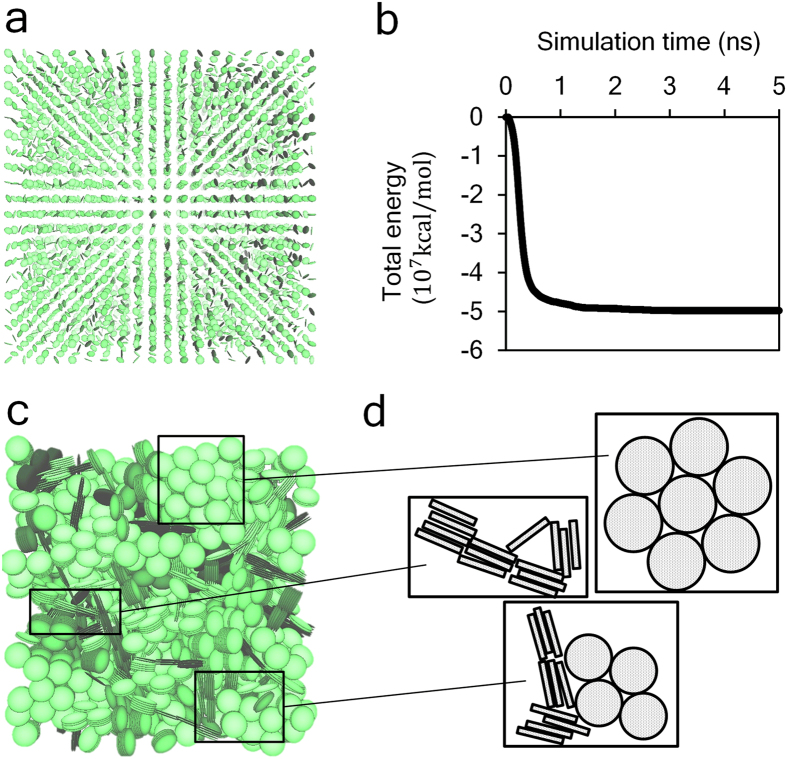
(**a**) Configuration of the coarse-grained C-S-H model at initial stage. (**b**) Change of total energy against simulation time. (**c**) Configuration of the equilibrated C-S-H system. (**d**) Three featured structures can be found in the modeled C-S-H system.

**Figure 4:**
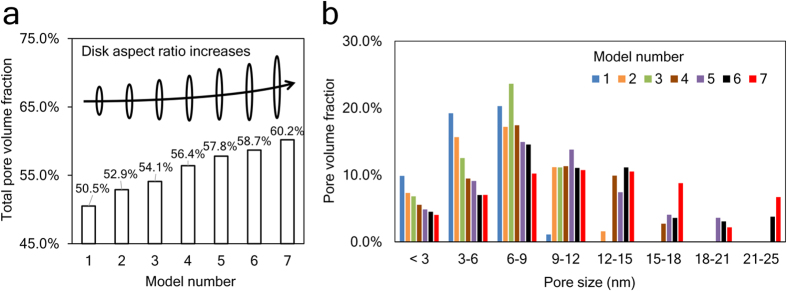
(**a**) Volume fraction of pore in the simulation box. The black bars over are schematics showing the lateral section of the disk-like C-S-H building blocks. The building blocks in these models are featured with varied diameter-to-thickness ratios. (**b**) Volume fraction of pores with different sizes.

Snapshot in Fig. 3a shows the configuration of the model with aspect ratio equal to 14.6. Figure 3b shows that the total energy of the system decreases to a stable value after 1-ns simulation, indicating an equilibrated state. The equilibrated trajectory shows randomly packed circles (front surfaces of a disk) and thin films (lateral section of a disk) from the side view as shown in Fig. 3c. The schematic drawings in Fig. 3d show three typical structures formed by those objects. Volume fraction of pores in the packed system is computed and displayed by Fig. 4a. It shows that the porosity of packed disks increases as the aspect ratio of the disk increases. The trend corresponds to what we have observed from nanoindentation tests, *i.e.*, C-S-H prepared at a higher w/c ratio contains more low-density phases and shows a higher porosity. The volume fraction of pores with different sizes is plotted in Fig. 4b. It shows that high-aspect-ratio disks tend to form large pores.

### Typical packing patterns of C-S-H building blocks

**Figure 5:**
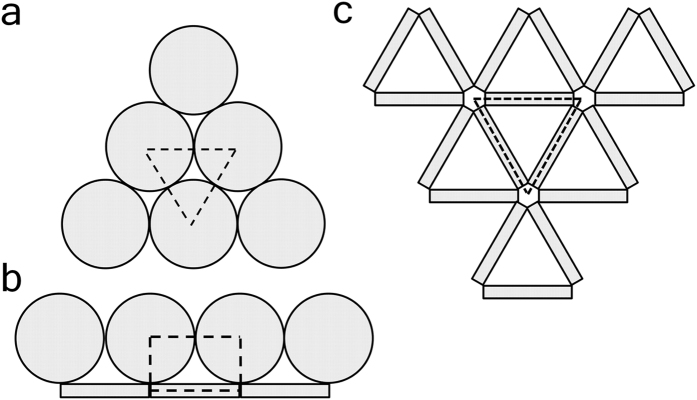
(**a–c**) The 3 typical structures, namely O, Ω and Δ shapes, formed by disk-like C-S-H building blocks. The dashed lines outline the repeating unit of the structure. From the repeating unit we can calculate the volume occupancy of particles. For O-type in case a, *η* = 0.907 is independent of diameter and thickness of the disk. For Ω-type in case b, 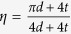 is slightly affected by the diameter (*d*) to thickness (*t*) ratio. For Δ-type in case c, 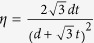 is highly related to the diameter to thickness ratio.

The packing of the disk-like objects is analyzed in 2-dimension space because 3-dimension structures can be learned as an integration of 2-dimension slices. Three characteristic pore structures are observed. Schematic diagram in Fig. 5a presents a bunch of circles, or cross sections of cylinder columns, representing a densely packed pattern in C-S-H. We use a letter **O** to denote such dense packing pattern. In **O**-type structure, the pore (area in between the tangent circles) size is smaller than 1 nm, corresponding to intra-globular pores. The dashed lines outline the repeating unit in **O**-type structure, which is composed of three tangent circles. The packing density of the unit is 0.907, independent of the aspect ratio of the C-S-H building block. The second type of structure is a combination of circles and thin films. We name this structure by a Greek letter **Ω** due to the resemblance in shape. As shown in Fig. 5b, this kind of structure is formed at the boundary between columns and lateral sections of disk-like objects. Size of the pores in **Ω**-shape ranges from 1 nm to 10 nm, corresponding to small gel pores. Packing density of the repeating unit in **Ω**-type structure is nearly independent of the aspect ratio of the C-S-H building block and the values range from 0.797 to 0.799, lower than HD (~0.85 in packing density) C-S-H phases. So we expect that HD C-S-H phase could be composed of **O-**type and **Ω-**type structures, while LD (~0.75 in packing density) should contain another loosely packed pattern. The third type of structure is formed by thin films, the lateral sections of the C-S-H building blocks shown in Fig. 5c. This structure is similar to a Greek letter **Δ**, indicating the simplest 2-dimension enclosure (*i.e.* a triangle) formed by pieces of thin films. The pore in the **Δ**-shape is larger than 10 nm, corresponding to capillary pores (*i.e.* 10 nm to 50 nm). The repeating unit in the **Δ**-shape structure is loosely packed and its packing density is a function of the diameter-to-thickness ratio. Packing density of this structure reduces from 0.190 to 0.166 among the models with increasing aspect ratio of disks (from 14.6 to 17.3 in model 1 to 7).

## Discussion

At nanoscale (smaller than 10 nm), the structural water is found to be a scaffolding component enhancing mechanical properties of C-S-H nanoparticles^13^. On the contrary, the sub-micron mechanical properties (characterized by nanoindentation with a detecting window of around 500 nm) are lower when water content increases^24^. This contradiction could originate at the transient scale^1^ (mesoscale, around 100 nm) between the nano- and the sub-micron scale. At the mesoscale, excess water content could influence the structure^23^ of flocculated C-S-H nanoparticles and degrade the mechanical properties.

In this paper, grid nanoindentation results show increasing amounts of low-density C-S-H phases and increasing porosity as w/c ratio increases from 0.3 to 0.66. This change of porosity could be related to the mesoscopic (around 100 nm) C-S-H structure, which is assumed to be composed of disk-like C-S-H building blocks. According to a SANS experiment^23^, the aspect ratio (diameter-to-thickness ratio) of C-S-H disk increases when water content increases. Following this observation, we simulate packing behaviors of rigid disks with varied aspect ratios. The simulations embody the nature of C-S-H by setting the shape of the disks close to C-S-H disks in reference to experiments^18^,^21^,^23^. Statistical analysis on pore size shows that high-aspect-ratio disks are likely to enclose large pores. The simulation is limited by a lack of information (such as polydispersity and compaction) about the C-S-H packing in real world so it must not be an exact reproduction of real C-S-H. However, the conceptual analysis on the packing patterns should be generic for similar colloidal systems composed of disk-like objects. Conceptually, we look at 3 typical packing patterns, including i) face-to-face columns, ii) face-to-side envelopes, and iii) side-to-side polygons, to demonstrate how aspect ratio influences pore formation. The side-to-side polygon shape features a packing density adaptive to the aspect ratio of C-S-H platelets, *i.e.*, more oblate platelets (with a higher aspect ratio) lead to a lower packing density. Combining the experimental observations and numerical simulations, we conclude that the increase of w/c ratio would increase the aspect ratio of C-S-H building blocks, enlarging the size and volume fraction of pores formed at the scale of around 100 nm.

## Methods

### Sample preparation

Cement pastes were prepared using ordinary Type I Portland cement. The water-to-cement (mass) ratio ranges from 0.3 to 0.7. It should be mentioned that for the 0.7 w/c ratio case, bleed water was accumulated and segregation was observed in cubic mold. In order to calculate the effective w/c ratio, we measured the density of cement after unmolding. The effective w/c ratio is 0.66 in the designed 0.7 w/c ratio case. Details of the sample preparation are provided in the supporting information.

### Nanoindentation test

The Triboindenter with a Berkovich tip (angle of 65.03°, tip radius of 0.2 μm) was used. A total of 100 indentations which were distributed as 10 × 10 grid were performed with 10 μm spacing length in each specimen. The designed loading program for C-S-H structure is as follows: 10 s for loading, 5 s for holding and 10 s for unloading stage. The 5 s holding time for peak load is designed to minimize the creep effect on the unloading^35^. Through applying continuum scale model to the *P-h* curve, two important quantities, *i.e.* hardness *H* and indentation modulus *E*_r_ can be calculated using equations (1) and (2) 36:


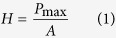



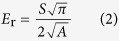


where *P*_max_ is the measured maximum indentation force; *A* is the projected contact area; *S* is the stiffness of unloading curve that can be evaluated based on 
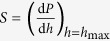
. A full set of nanoindentation data is provided in supporting information.

### Statistical analysis on nanoindentation data

After obtaining a matrix of *(H, M)* data points, we adopt the following statistical approaches to extract information about packing density (*η*). For details of these formulas of the two functions in equation (3), the reader is kindly referred to the supporting information as well as literature^25^.


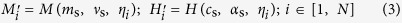


Here, *N* is the number of data points, {*m*_s_, *v*_s_} are stiffness and Poisson’s ratio of mineral phase in C-S-H, {*c*_s_, *α*_s_} are cohesion and friction coefficient ookf the C-S-H mineral, which are to be understood in the sense of the Drucker-Prager strength model^25^. Unknowns {*m*_s_, *v*_s_, *c*_s_, *α*_s_, *η*_*i*_}, *i* ∈ [1, *N*] are determined by minimizing the difference between calculation and experimental results, as shown in equation (4). All the *(H, M, η)* data points are listed in supporting information. The minimization is performed with home-made MATLAB codes and the details are listed in supporting information.


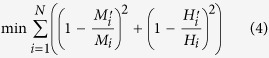


Now we have a collection of *(H, M, η)* data. Each *(H, M, η)* data point falls into one of four characteristic compositions, namely loosely-packed (LP), low-density (LD), high-density (HD) and ultra-high-density (UHD) C-S-H. As a result, the entire *(H, M, η)* data collection, represented by a cumulative distribution function (CDF), is a combination of 4 sub-functions. Experimental CDF is obtained by counting data points as defined by equation (5). Theoretically, the CDF can be deconvoluted into 4 sub-functions as shown in equation (6). These sub-functions are in form of Gaussian CDF with mean value *μ*, standard deviation *s* and phase fraction *f*.


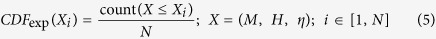



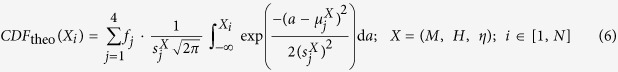


The next task, deconvolution, is to minimize the difference between theoretical and experimental CDF. The minimization is achieved by adapting 28 unknowns 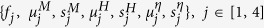 subjected to two constraints, as shown in equation (7). The minimization is performed with home-made MATLAB codes and the details are listed in supporting information.


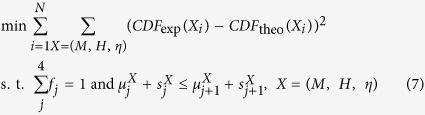


We obtain fraction of the four characteristic C-S-H phases from the deconvolution results. Finally, the gel porosity is calculated by equation (8).


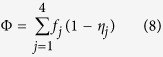


### Molecular dynamics simulation

The fundamental assumption for developing coarse-grained C-S-H models is the concept of disk-like building blocks^11^,^30^, which have been identified in recent experimental works^18^,^23^,^37^. It has been found that disk-like objects with high aspect ratio (diameter-to-thickness ratio) exist in C-S-H. The C-S-H in cement paste prepared at higher w/c ratios consists of larger disk-like building blocks. Hence, we set up a series of models with increasing aspect ratios for representing the increasing trend of w/c ratio. The diameter is from 3.25 nm to 4.75 nm, according to colloidal model-II (CM-II)^18^,^21^. The aspect ratio increases from 14.6 to 17.3, corresponding to the SANS experiment^23^. The interaction between building blocks is described by the Gay-Berne potential^38^, which calculates potential energy between pairwise spheroids, with considerations of shape, rotation and position of each particle. It has been employed to simulate clay minerals with an oblate shape^39^ and disk-like C-S-H building blocks as well^11^,^30^,^31^. The key idea is that when the aspect ratio of an ellipsoid is large (above 10), the ellipsoid resembles a disk. The potential energy of a pair of platelets is calculated by equation (9).


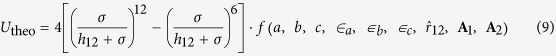


Detailed formula of the function in equation (9) can be found in literature^39^ and supporting information. Unknown parameters {*∈*_*a*_, *∈*_*b*_, *∈*_*c*_}, which determine the values of energy well depth, can be derived from a minimization process, as shown by equation (10).


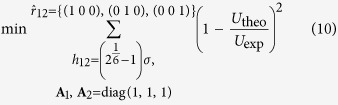


The *U*_exp_ is the adhesion energy of C-S-H, calculated by multiplying the normalized surface energy by the surface area, *e.g.*, in the face-to-face case 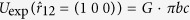, where *G*~450 mj/m^2^ is characterized by both experiments and atomistic simulations^11^,^40^,^41^. After defining the parameters for GB potential, we perform molecular dynamics simulations. The initial coarse-grained (CG) model is set up by averagely distributing the 8000 (20 × 20 × 20) beads in a simulation box, with a 6-nm spacing. The cutoff of GB potential is set to be 6.25 nm. With a 1 fs time step, the system is equilibrated for 20 ns in NPT ensemble with temperature and pressure controlled at 300 K and 1 atm respectively. The equilibrated trajectory is analyzed statistically to obtain porosity and pore size distribution. Details about the minimization process, the parameters and the statistical analysis can be found in supporting information.

## Additional Information

**How to cite this article**: Yu, Z. *et al.* Mesoscopic packing of disk-like building blocks in calcium silicate hydrate. *Sci. Rep.* **6**, 36967; doi: 10.1038/srep36967 (2016).

**Publisher’s note**: Springer Nature remains neutral with regard to jurisdictional claims in published maps and institutional affiliations.

## Supplementary Material

Supplementary Information

Supplementary Dataset 1

## Figures and Tables

**Table 1:** Deconvolution results of nanoindentation tests on cement specimens with varied w/c ratio.

Specimen	w/c	0.3	0.4	0.5	0.6	0.7 (0.66)
Phase1	*f*	4%	7%	8%	37%	35%
Loosely-packed (LP) C-S-H	*M* (GPa)	10.01 ± 1.51	9.78 ± 2.39	10.74 ± 2.50	11.33 ± 3.69	10.14 ± 3.36
	*H* (GPa)	0.11 ± 0.11	0.39 ± 0.07	0.35 ± 0.02	0.33 ± 0.14	0.16 ± 0.07
	*η*	0.56 ± 0.05	0.56 ± 0.05	0.60 ± 0.02	0.60 ± 0.04	0.57 ± 0.03
Phase2	*f*	26%	33%	34%	36%	41%
Low-density (LD) C-S-H	*M* (GPa)	20.71 ± 3.74	18.36 ± 4.44	18.49 ± 4.95	19.42 ± 4.40	19.51 ± 3.67
	*H* (GPa)	0.44 ± 0.15	0.55 ± 0.09	0.52 ± 0.11	0.60 ± 0.14	0.60 ± 0.19
	*η*	0.65 ± 0.04	0.64 ± 0.03	0.65 ± 0.03	0.66 ± 0.02	0.66 ± 0.04
Phase3	*f*	54%	49%	40%	20%	15%
High-density (HD) C-S-H	*M* (GPa)	30.37 ± 5.92	28.52 ± 5.72	30.45 ± 6.00	30.29 ± 3.50	30.39 ± 3.11
	*H* (GPa)	0.80 ± 0.21	1.03 ± 0.30	1.04 ± 0.20	0.98 ± 0.14	1.11 ± 0.32
	*η*	0.73 ± 0.04	0.72 ± 0.05	0.76 ± 0.04	0.74 ± 0.03	0.75 ± 0.04
Phase4	*f*	16%	11%	19%	7%	9%
Ultra-high-density (UHD) C-S-H	*M* (GPa)	49.53 ± 3.60	49.34 ± 3.71	49.62 ± 4.37	49.96 ± 3.04	49.99 ± 3.04
	*H* (GPa)	1.51 ± 0.21	1.98 ± 0.25	1.76 ± 0.27	1.62 ± 0.50	2.11 ± 0.30
	*η*	0.82 ± 0.03	0.87 ± 0.04	0.87 ± 0.04	0.78 ± 0.01	0.85 ± 0.03
Porosity	Φ	28% ± 4%	30% ± 4%	27% ± 4%	34% ± 3%	34% ± 3%

*M*, *H* and *η* denote indentation hardness, modulus and packing density respectively. The porosity at the last line is calculated by 
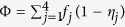
.
