# Supplementary material for: Mesoscopic packing of disk-like building blocks in calcium silicate hydrate
Source: Sci Rep. 2016 Nov 15;6:36967. doi: 10.1038/srep36967 (PMC5109495; doi:10.1038/srep36967)
Supplement: Supplementary Information [file srep36967-s1.pdf]

Supporting Information

Mesoscopic packing of disk-like building blocks in calcium silicate hydrate

Zechuan Yu[a], Ao Zhou[a] and Denvid Lau[a,b,*]

[a] Department of Architecture and Civil Engineering, City University of Hong Kong, Hong Kong, China

[b] Department of Civil and Environmental Engineering, Massachusetts Institute of Technology, Cambridge, MA 02139, USA

* Corresponding author: denvid@mit.edu

Sample preparation

Cement paste was prepared using ordinary Type I Portland cement. The strength class of the Portland cement is 52.5 N and Blaine fineness of cement is 358 m^2/kg in accordance with the Standard BS EN 197-1:2000. The composition of the cement is shown in Table S1. The water-to-cement (mass) ratio ranges from 0.3 to 0.7. Accordingly, the cement paste was prepared by mixing cement powdery with the corresponding tab water in weight. Then the mixture is poured into the cubic molds of length 30 mm and vibrated for 30 s. After 24 hours, the cement paste was unmolded and put into water tank for 28 days at 20 °C for curing. After curing, the cement paste was cut into 10 × 10 × 8 mm block with diamond saw. For the case in which the w/c ratio is 0.7, bleed water was accumulated and segregation was observed in cubic mold. In order to have a more precise evaluation of 0.7 w/c ratio case, the effective mass density of cement paste right after unmolding was measured and recorded as 1.695 g/cm^3. For the designed 0.7 w/c ratio case, the effective w/c ratio right after unmolding is 0.66.

Table S1 The composition of ordinary Type I Portland cement (in mass). (from manufacturer)

Composition	CaO	SiO_2	Al_2O_3	Fe_2O_3	SO_3	MgO	K_2O	others
Percentage (%)	64.3	19.7	5.1	2.8	2.6	1.5	0.6	3.4

In order to do the characterization of morphology and mechanical properties at nanoscale, a smooth sample surface should be prepared. All surfaces were ground by silicon carbide paper with grade

220, 400, 600, 800, 1000 and 1200 sequentially. Then the samples were polished on a low-relief polishing cloth with increasing fineness of diamond paste. Finally, the samples were put into ethanol for ultrasonic bath cleaning for 10 min to remove the impurities and dust left on the surface. After preparation, all samples were well stored in a container to confirm the surface quality.

Scanning electron microscope investigation

Scanning electron microscope (SEM) is used for observing the surface of cement paste. The microstructure of cement paste can be observed under the FEI Quanta 450 FEG SEM. Except aforementioned sample preparation process, the cement paste sample for SEM should be coated with gold (Au) before SEM examination to make the surface of sample conductive. During the SEM examination, low voltage was used and working distance was 13 mm.

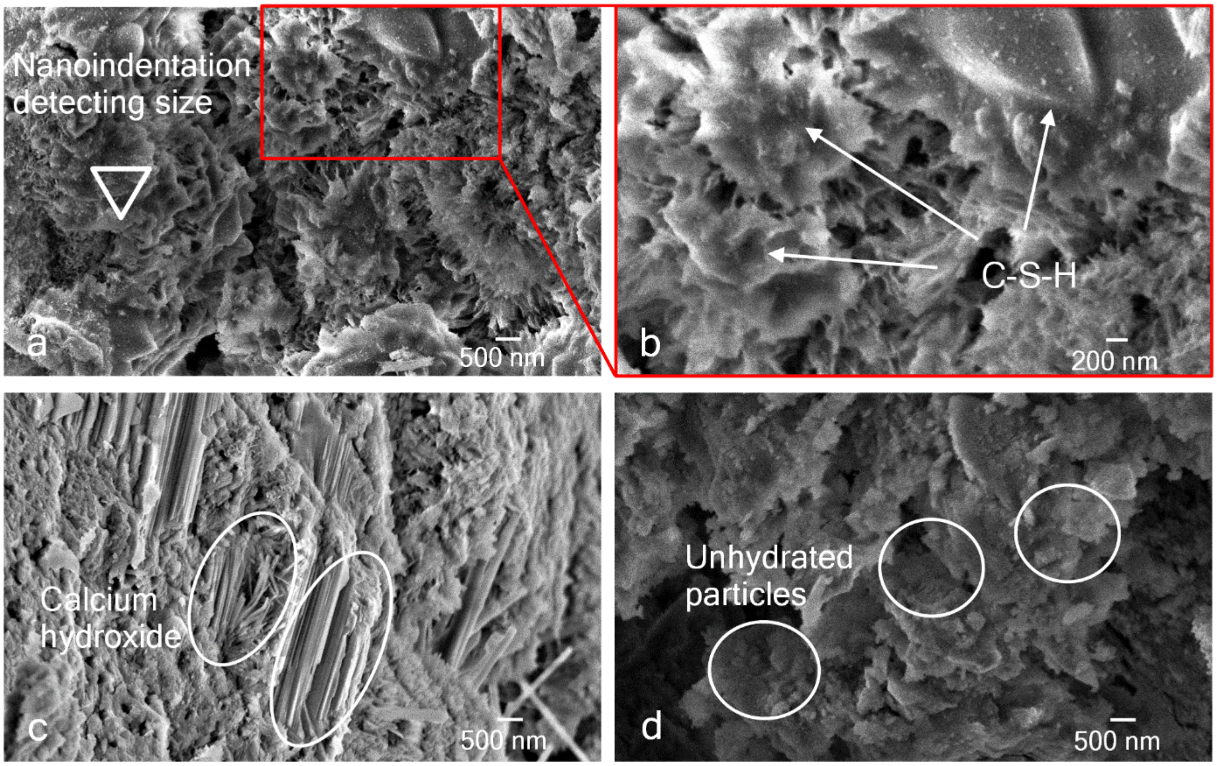

Figure S1 SEM images of cement paste samples with magnification of **a** ×30,000 **b** ×70,000 **c** ×30,000 **d** ×30,000. Three regions, with the same size of the indenter used in the nanoindentation tests, are marked out as shown in Figure S1a. The detecting window size of nanoindentation is outlined by a triangle

Indentation hardness and indentation modulus data

Nanoindentation test data points in (H, M, η) pairs. The unit of (H, M) is GPa. The relationship between η and (H, M) is shown in appendix A2 and A3. The corresponding water-to-cement (w/c) ratios to set 1, set 2, set 3-1, set 3-2, set 4 and 5 are 0.3, 0.4, 0.5, 0.5, 0.6, 0.7, respectively. The '/' data point indicates an uninterpretable nanoindentation point. See the separate Excel document provided online.

Detailed formula of Eq. (3) for relating packing density with indentation modulus and indentation hardness

From packing density to indentation modulus

$$M_i' = M(m_\mathrm{s}, \nu_\mathrm{s}, \eta_i) = m_\mathrm{s} \cdot g \frac{(9\eta_i r_\mathrm{s} + 4g + 3r_\mathrm{s})(3r_\mathrm{s} + 4)}{4(4g + 3r_\mathrm{s})(3r_\mathrm{s} + 1)},$$

$$\text{where } r_s = \frac{2(1 + \nu_\mathrm{s})}{3(1 - 2\nu_\mathrm{s})}$$

$$\text{and } g = \frac{1}{2} - \frac{5}{4}(1 - \eta_i) - \frac{3}{16} r_\mathrm{s}(2 + \eta_i)$$

$$+ \frac{1}{16}\sqrt{144(1 - r_\mathrm{s}) - 480\eta_i + 400\eta_i^2 + 408 r_\mathrm{s}\eta_i - 120 r_\mathrm{s}\eta_i^2 + 9r_\mathrm{s}^2(2 + \eta_i)^2}$$

The subscript "s" denotes "solid". From packing density to indentation hardness

$$H_i' = H(c_\mathrm{s}, \alpha_\mathrm{s}, \eta_i) = c_\mathrm{s} \cdot \Pi \cdot [1 + (1 + \eta_i)\alpha_\mathrm{s} - (d - e\eta_i)\alpha_\mathrm{s}^2 - (f - g\eta_i)\alpha_\mathrm{s}^5],$$

$$\text{where } \Pi = \frac{12\eta_i(a - b\eta_i)\sqrt{(2\eta_i - 1)(2 + \eta_i)}}{(1 - c\eta_i)(2 + \eta_i)}$$

and a=0.19567, b=0.03739, c=0.77999, d=20.3138, e=31.5352, f=52.1817, g=99.3465

Fitted mineral properties $(\boldsymbol{m}_\mathbf{s}, \boldsymbol{\nu}_\mathbf{s}, \boldsymbol{c}_\mathbf{s}, \boldsymbol{\alpha}_\mathbf{s})$ calculated from each data set and the optimization error

C-S-H Mineral properties	Set 1	Set 2	Set 3-1&2	Set 4	Set 5
Stiffness m_s/GPa	64.2	62.5	63.3	62.5	62.4
Poisson's ratio ν_s	0.002	0.213	0.386	0.421	0.033
Cohesion c_s/GPa	0.248	0.497	0.488	0.327	0.284
Friction Coefficient α_s	0.382	0.194	0.120	0.324	0.381
Packing density error	5.744	5.298	9.714	10.49	6.968
Relative error of M, $\bar{\mathrm{e}}_M$	-4.6%	-3.4%	-4.3%	-7.2%	-1.8%
Standard deviation, $\bar{\bar{\mathrm{e}}}_M$	22.1%	17.8%	19.3%	25.1%	22.4%
Relative error of H, $\bar{\mathrm{e}}_H$	-1.4%	-3.7%	-3.4%	-3.7%	-3.2%
Standard deviation, $\bar{\bar{\mathrm{e}}}_H$	10.6%	18.6%	17.3%	19.1%	13.9%
Deconvolution error	0.0012	0.0007	0.0006	0.0008	0.0005

$$\text{Packing density error} = \sum_{i=1}^{N}\left(\left(1-\frac{M_i'}{M_i}\right)^2+\left(1-\frac{H_i'}{H_i}\right)^2\right)$$

$$\text{Relative error}, \bar{\mathrm{e}}_X = \frac{1}{N}\sum_{i=1}^{N}\mathrm{e}_{X,i}\text{, where } \mathrm{e}_{X,i} = \left(\frac{X_i'-X_i}{X_i}\right) \text{ and } X = (M,H)$$

$$\text{Standard deviation of relative error}, \bar{\bar{\mathrm{e}}}_X = \sqrt{\frac{1}{N-1}\sum_{i=1}^{N}\left(\mathrm{e}_{X,i}-\bar{\mathrm{e}}_X\right)^2}\text{, where } X = (M,H)$$

$$\text{Deconvolution error} = \sum_{i=1}^{N}\sum_{X=(M,H,\eta)}\left(CDF_{\mathrm{exp}}(X_i)-CDF_{\mathrm{theo}}(X_i)\right)^2$$

For parameters set $(m_s, \nu_s, c_s, \alpha_s)$, the initial values are (63, 0.20, 0.35, 0.25), lower bound and upper bound are (50, 0.00, 0.20, 0.10) and (100, 0.50, 0.50, 0.40), respectively. The optimization is performed using MATLAB function 'fmincon' with the algorithm option 'sqp'.

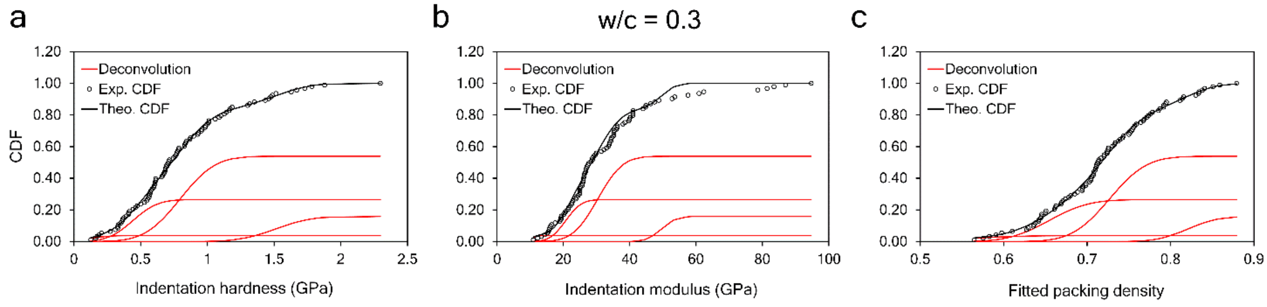

Figure S2 a b c Fitting curves of set 1 data (w/c=0.3) in the dimension of indentation hardness, indentation modulus and packing density respectively

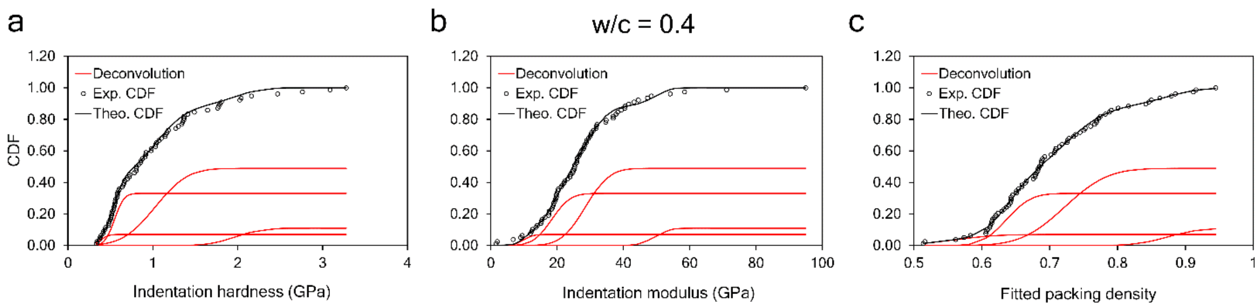

Figure S3 a b c Fitting curves of set 2 data (w/c=0.4) in the dimension of indentation hardness, indentation modulus and packing density respectively

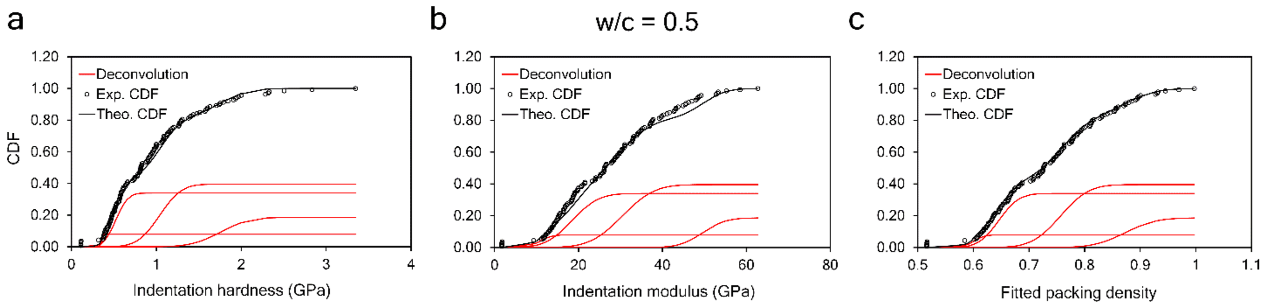

Figure S4 a b c Fitting curves of set 3 data (w/c=0.5) in the dimension of indentation hardness, indentation modulus and packing density respectively

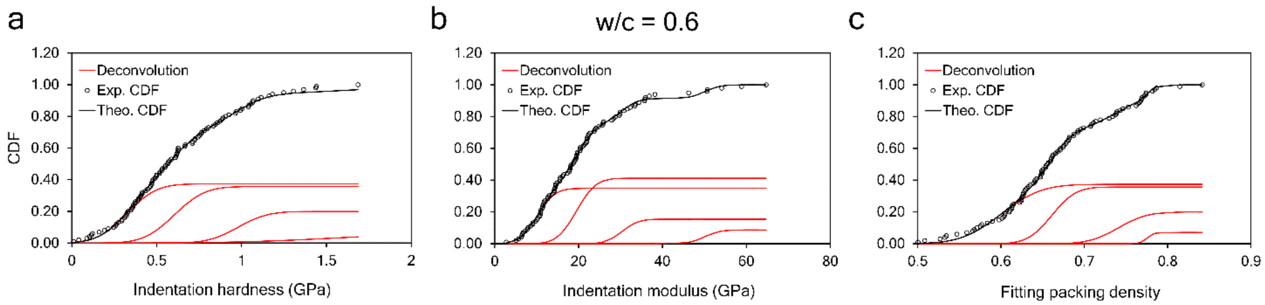

Figure S5 a b c Fitting curves of set 4 data (w/c=0.6) in the dimension of indentation hardness, indentation modulus and packing density respectively

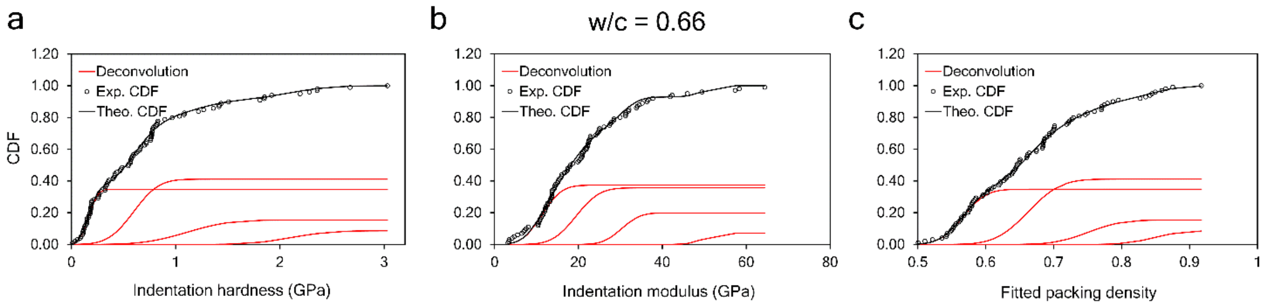

Figure S6 a b c Fitting curves of set 5 data (w/c=0.66) in the dimension of indentation hardness, indentation modulus and packing density respectively

Detailed formula of Eq. (9) for GB potential and optimization error

$$U_{\text{theo}} = 4\left[\left(\frac{\sigma}{h_{12}+\sigma}\right)^{12} - \left(\frac{\sigma}{h_{12}+\sigma}\right)^{6}\right] \cdot f(a,b,c,\epsilon_a,\epsilon_b,\epsilon_c,\hat{\mathbf{r}}_{12},\mathbf{A}_1,\mathbf{A}_2),$$

$$\text{where } f(a,b,c,\epsilon_a,\epsilon_b,\epsilon_c,\hat{r}_{12},\mathbf{A}_1,\mathbf{A}_2) = \eta_{12}(a,b,c,\mathbf{A}_1,\mathbf{A}_2) \cdot \chi_{12}(\epsilon_a,\epsilon_b,\epsilon_c,\hat{r}_{12},\mathbf{A}_1,\mathbf{A}_2)$$

$$h_{12} = r - \left(\frac{1}{2}\hat{\mathbf{r}}_{12}^T\mathbf{G}_{12}^{-1}\hat{\mathbf{r}}_{12}\right)^{-\frac{1}{2}}, \mathbf{G}_{12} = \mathbf{A}_1^T\mathbf{S}^2\mathbf{A}_1 + \mathbf{A}_2^T\mathbf{S}^2\mathbf{A}_2 \text{ and } \mathbf{S} = \text{diag}(a,b,c)$$

$$\eta_{12} = \left[\frac{2s^2}{\det(\mathbf{G}_{12})}\right]^{\frac{1}{2}} \text{ and } s = (ab+cc)(ab)^{\frac{1}{2}}$$

$$\chi_{12} = 2\hat{\mathbf{r}}_{12}^T\mathbf{B}_{12}^{-1}\hat{\mathbf{r}}_{12} \text{ and } \mathbf{B}_{12} = \mathbf{A}_1^T\mathbf{E}^{-1}\mathbf{A}_1 + \mathbf{A}_2^T\mathbf{E}^{-1}\mathbf{A}_2$$

Input parameters include

- $a, b, c,$ the three radii of ellipsoids and $\sigma = \min(a,b,c)$
- $\epsilon_a, \epsilon_b, \epsilon_c,$ the relative well depths in three radial directions
- $\vec{r}_1, \vec{r}_2,$ the position vectors of the center of two ellipsoids, $r = \|\vec{r}_2 - \vec{r}_1\|, \hat{\mathbf{r}}_{12} = \frac{\vec{r}_2 - \vec{r}_1}{r}$
- $\mathbf{A}_1, \mathbf{A}_2,$ the 3×3 rotation matrices of two ellipsoids

The relative well depths, $\epsilon_a, \epsilon_b, \epsilon_c$ are fitted against the empirical values of the potential energy. The term h_{12} is the closest distance between two ellipsoids, r is the center-to-center distance. Analytically, the minimal potential energy, or the energy well depth, which is referred to as the adhesion energy of C-S-H, is obtained when $h_{12} = \left(2^{\frac{1}{6}} - 1\right)\sigma$. As a result, this criterion is required in the parameterization process. Values of the parameters are listed in Table S2.

The parameters $\mathbf{A}_1$ and $\mathbf{A}_2$ are 3-dimension rotation matrices that describe the rotation of a pair of disk-like object. When defining the parameters, the rotation matrices are set to identity matrices for simplification. Because translating operations are adequate to manipulate the pair of disks for creating face-to-face and side-to-side alignments.

Table S2 GB potential parameters. The parameters *a*, *b* and *c* are three radii of the disk-like build blocks. Other three parameters are relative potential energy well depths, which are derived from the minimization process. The error is a product out of Eq. (10).

Model	*2a* (nm)	*2b=2c* (nm)	Aspect ratio	$\epsilon_a(10^{-21}\text{J})$	$\epsilon_b = \epsilon_c\ (10^{-21}\text{J})$	error
1	0.22250	3.25	14.6	1827.4	159.4	3.9×10^{-10}
2	0.23125	3.50	15.1	2086.7	175.6	5.3×10^{-10}
3	0.24000	3.75	15.6	2362.2	192.6	6.9×10^{-10}
4	0.24875	4.00	16.1	2653.9	210.2	9.0×10^{-10}
5	0.25750	4.25	16.5	2961.7	228.6	1.1×10^{-9}
6	0.26625	4.50	16.9	3285.6	247.6	1.4×10^{-9}
7	0.27500	4.75	17.3	3625.6	267.4	1.9×10^{-9}

Statistical analysis on pore distribution in the 3-d model

The packing system of disk-like objects is digitalized into a cubic 3-d matrix containing 0 or 1 values, where 0 indicates void and 1 indicates solid. The resolution is 0.25 nm and the cubic matrix dimension is 149, 160, 170, 182, 192, 202 and 214 for model 1, 2, 3, 4, 5, 6 and 7, respectively. On the basis of the 0-1-value matrix, we develop a marking algorithm that grades the void points to different levels. Starting from the solid points, adjacent void points are graded by 2. Next, the void points in the vicinity of level 2 points are marked by 3. Progressively, the entire space (digital 3-d matrix) with be marked with integers starting from 1. The marked matrix contains values 1, 2, 3 …, where 1 indicates solid and the following values indicate void with different levels. The higher the level is, the 'deeper' the void is. The maximum void level can indicate the size of the largest pore in the packing system. Typical distribution of the values in a cross section is shown by a contour plot in Figure S7. The volume fraction (f_{void}(level)) characterized at the different void levels is shown in Table S3. Because the solid part in this model represents the solid C-S-H, the spots in the 0.25-nm vicinity (marked by level 2) could represent the intra-particle and surface-absorbed water molecules. As a result, level 1 and level 2 spots together could represent the fully saturated C-S-H nanoparticles with a density ($\rho_{\text{nanoparticle}}$) of approximately 2470 kg/m^3 (H=2.1 case in [1]). Assuming that all other pores are filled with water, the density of the entire system, the packing density (η) and the porosity (Φ) of the model can be calculated using the equation below.

$$\eta = \frac{(1 - f_{\text{void}}(3)) \cdot \rho_{\text{nanoparticle}} + f_{\text{void}}(3) \cdot \rho_{\text{water}}}{\rho_{\text{nanoparticle}}} \text{ and } \Phi = 1 - \eta$$

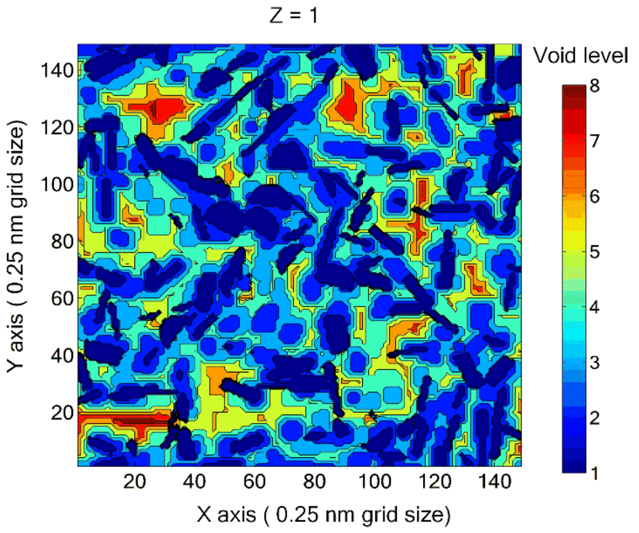

Figure S7 Cross-section contour plot of the distribution of void levels in model 1 at Z=1 plane. Level 1 spots are solid C-S-H. Level 2 spots represent smallest gel pores. Higher levels correspond to larger gel pores

Table S3 Void volume fraction at increasing void levels in different models. The volume fraction is the calculated as the ratio between the number of spots with void level higher than or equal to the level and the total number of spots.

Model	Level1	Level2	Level3	Level4	Level5	Level6	Level7	Level8	Higher
1	100%	69.7%	50.5%	30.7%	13.8%	4.6%	1.1%	0.2%	0.0%
2	100%	70.6%	52.9%	34.3%	17.5%	7.0%	2.2%	0.6%	0.1%
3	100%	70.3%	54.1%	36.9%	20.4%	9.4%	3.7%	1.2%	0.4%
4	100%	71.4%	56.4%	40.1%	23.6%	11.8%	5.0%	1.9%	0.6%
5	100%	71.7%	57.8%	42.5%	26.4%	14.1%	6.6%	2.6%	0.9%
6	100%	71.8%	58.7%	44.3%	28.6%	16.4%	8.4%	3.9%	1.7%
7	100%	72.3%	60.2%	46.7%	31.3%	18.6%	9.8%	4.6%	2.0%

On the graded 3-d matrix, we employ a breadth-first search (BFS) algorithm to find connected clusters with the void level higher than 2. Each cluster represents a single enclosed pore. The size of the pore is calculated as the diameter of an equivalent-volume sphere. Distribution of volume fraction of pores with different sizes is displayed in Table S4.

Table S4 Volume fraction of pores with equivalent-sphere diameter from 3 nm to 24 nm with 3-nm step size. The minimum pore size is 0.43 nm and the maximum is 24.4 nm found in model 7.

Model	<3	3-6	6-9	9-12	12-15	15-18	18-21	21-24	Total
1	9.9%	19.2%	20.3%	1.1%	0	0	0	0	50.5%
2	7.3%	15.7%	17.2%	11.2%	1.5%	0	0	0	52.9%
3	6.8%	12.6%	23.6%	11.1%	0	0	0	0	54.1%
4	5.6%	9.5%	17.4%	11.3%	9.9%	2.7%	0	0	56.4%
5	4.9%	9.1%	14.9%	13.8%	7.4%	4.1%	3.6%	0	57.8%
6	4.5%	7.0%	14.5%	11.1%	11.1%	3.6%	3.1%	3.8%	58.7%
7	4.0%	7.0%	10.2%	10.7%	10.5%	8.8%	2.2%	6.8%	60.2%

Supporting information references

1 Jennings, H. M. Refinements to colloid model of C-S-H in cement: CM-II. *Cement and Concrete Research* **38**, 275-289, doi:http://dx.doi.org/10.1016/j.cemconres.2007.10.006 (2008).
